# Production and characterization of immunoglobulin G anti-rLipL32 antibody as a biomarker for the diagnosis of leptospirosis

**DOI:** 10.14202/vetworld.2024.871-879

**Published:** 2024-04-19

**Authors:** Susanti Susanti, Pratiwi Pudjilestari Sudarmono, N. L. P. Indi Dharmayanti, Prasandhya Astagiri Yusuf

**Affiliations:** 1 Doctoral Program in Biomedical Sciences, Faculty of Medicine, Universitas Indonesia, Jakarta, 10430, Indonesia; 2 Department of Microbiology, Faculty of Medicine, Universitas Indonesia, Jakarta, 10430, Indonesia; 3 Research Center for Veterinary Science, Research Organization for Health, National Research and Innovation Agency, Bogor, 16911, Indonesia; 4 Medical Physiology Biophysics Department and Medical Technology IMERI, Faculty of Medicine, Universitas Indonesia, Jakarta, 10430, Indonesia

**Keywords:** anti-rLipL32 serum, immunoglobulin G anti-rLipL32 antibody, *Leptospira*, rLipL32 protein

## Abstract

**Background and Aim:**

Microscopic agglutination test (MAT) for the diagnosis of leptospirosis requires live cultures and is serovar-specific, while polymerase chain reaction (PCR) requires expensive equipment and sample preparation. The rLipL32 protein is conserved and can be used for the production of immunoglobulin G (IgG) anti-rLipL32 antibody, which can be used as a biomarker for leptospirosis diagnosis. This study aimed to produce and characterize an IgG anti-rLipL32 antibody as a biomarker for leptospirosis diagnosis.

**Materials and Methods:**

*Escherichia coli* rLipL32 was cultured and analyzed by PCR and sequencing. Cultures were used for rLipL32 protein expression and purification and the rLipL32 protein was analyzed by sodium dodecyl sulfate-polyacrylamide gel electrophoresis (SDS-PAGE). The rLipL32 protein was used to produce anti-rLipL32 serum and was analyzed by enzyme-linked immunosorbent assay (ELISA). Serum was purified to obtain IgG anti-rLipL32 antibody and characterized by SDS-PAGE and western blotting.

**Results:**

PCR was able to amplify the LipL32 gene from *E. coli* rLipL32, and sequencing analysis showed 99.19% similarity with pathogenic *Leptospira*. SDS-PAGE analysis showed a 32-kDa band. ELISA results showed an increase in OD in anti-rLipL32 serum compared to preimmune serum. Western blotting results showed that the IgG anti-rLipL32 antibody was able to bind and cross-reacts with pathogenic *Leptospira* serovar but not with *E. coli* or *Staphylococcus aureus*.

**Conclusion:**

IgG anti-rLipL32 antibody has high specificity and sensitivity against *Leptospira* pathogens. These findings suggest that IgG anti-rLipL32 antibody is a promising biomarker for the diagnosis of leptospirosis.

## Introduction

Leptospirosis is a global zoonotic disease caused by pathogenic spirochetes from the genus *Leptospira* [1], which is prevalent in tropical and subtropical areas [2]. *Leptospira* can infect mammals and rodents and act as the main carrier of *Leptospira* [3]. Bacteria are excreted in the urine of the infected animals and pollute the environment. Infection in humans can occur through direct or indirect contact with infected animals [4]. Leptospirosis cases in humans and leptospirosis seroprevalence in animals are currently increasing, and many cases have not been detected due to difficulties in diagnosing leptospirosis and limited laboratories in Indonesia. Early detection of disease-causing agents is critical for clinical and treatment purposes; therefore, precise and accurate detection is urgently needed [5–8]. Leptospirosis is often underdiagnosed because its symptoms are similar to those of other infectious diseases, such as dengue fever and malaria. Therefore, a laboratory diagnosis is very necessary [9–12].

Diagnosis of pathogenic *Leptospira* relies on different laboratory tests, such as detection of specific antibodies by microscopic agglutination test (MAT) or enzyme-linked immunosorbent assay (ELISA). *Leptospira* or its components can also be detected in urine or tissues by darkfield microscopy, culture, polymerase chain reaction (PCR), or immunostaining [13]. Culture of *Leptospira* requires specific media and a long time due to its slow growth rate, which results in delayed diagnosis and low sensitivity [14]. MAT is the gold standard serological test for the diagnosis of leptospirosis; however, it is quite complex, serovar-specific, and requires a specialized laboratory for live *Leptospira* serovar cultures [15, 16]. Antigens representing all known serogroups or strains should be used to obtain optimal sensitivity [17, 18]. Molecular-based detection usually requires skilled personnel, high analysis costs, expensive instruments, and sample preparation, which makes it difficult to implement in the field [19, 20]. Modern serological tests for diagnosing leptospirosis have been developed using bacterial recombinant antigens [21]. At present, leptospiral outer membrane proteins (OMPs) are widely used for developing leptospirosis diagnostic tests. These OMPs play critical roles in leptospiral pathogenesis [22]. The OMPs of *Leptospira* are divided into three classes: Lips (lipoproteins), including LipL42, LipL32, and LipL24; transmembrane proteins, including OmpL1; and peripheral proteins, including LipL42 [23]. LipL32 is an outer membrane lipoprotein of pathogenic *Leptospira*, measuring 32-kDa, expressed at high levels during infection, highly conserved, and the most abundant lipoprotein in *Leptospira interrogans* [21, 24–27]. This protein can be used as a diagnostic antigen in patients with acute and convalescent leptospirosis [28]. This protein can also be used for the production of an immunoglobulin G (IgG) anti-rLipL32 antibody as a biomarker for developing valuable diagnostic tools.

This study aimed to produce and characterize an IgG anti-rLipL32 antibody as a biomarker for developing leptospirosis diagnostic techniques. The novelty of this study is that the rLipL32 protein was used to produce anti-rLipL32 serum in rabbits using the adjuvant Montanide ISA 70 MVG (Seppic, Paris, France) compared with Freund’s adjuvant and analyzed using in-house ELISA. The produced anti-rLipL32 serum was then used for the production of anti-rLipL32 IgG and characterized by sodium dodecyl sulfate-polyacrylamide gel electrophoresis (SDS-PAGE) and western blotting.

## Materials and Methods

### Ethical approval

The production of *Leptospira* antiserum in rabbits was performed in accordance with the guidelines for the use of laboratory animals based on animal welfare principles. All procedures were approved by Balitbangtan/BB Litvet/Rd_Rm/01.01/2022 Animal Welfare Committee of the Indonesian Agency for Agricultural Research and Development.

### Study period and location

This study was conducted from April 2022 to April 2023 at the Balai Besar Pengujian Standar Instrumen Veteriner, Bogor, Indonesia; the Genomics Laboratory of the National Research and Innovation Agency, Bogor, Indonesia; and the Department of Microbiology, Faculty of Medicine, Universitas Indonesia, Jakarta, Indonesia.

### *Leptospira, Escherichia coli*, and *Staphylococcus aureus* bacterial culture

*Leptospira* serovar hardjo, icterohaemorrhagiae, bataviae, javanica, ballum, celledoni, pyrogenes, canicola, cynopteri, pomona, rachmati, australis, grippotyphosa, and tarassovi were obtained from Koninklijk Institut Voor de Tropen, Amsterdam, The Netherlands. *E. coli* and *S. aureus* were obtained from Bacteriology Laboratory, Balai Besar Penelitian Veteriner. *Leptospira* bacteria were cultured in Ellinghausen–McCullough–Johnson–Harris media and incubated at 30°C for 5–7 days until the antigen concentration was approximately 1.5 × 10^8^ cells/mL. *E. coli* and *S. aureus* were cultured on nutrient agar media and incubated overnight at 37°C. Bacteria were then collected into a suspension equivalent to 0.5 McFarland (1.5 × 10^8^ cells/mL). These three bacteria were used for western blotting analysis of IgG anti-LipL32 antibody.

### *E. coli* BL21 (DE3) pLysS containing pRSET C-LipL32 bacterial culture

*E. coli* BL21 (DE3) pLysS containing pRSET C-LipL32 was grown overnight at 37°C in Luria-Bertani (LB) agar containing 35 μg/mL chloramphenicol and 50 μg/mL ampicillin. Bacterial colony growth was confirmed by PCR and sequencing. In addition, this culture was used for the rLipL32 protein expression stage.

### Analysis of *E. coli* BL21 (DE3) pLysS cultures containing pRSET C-LipL32 by PCR analysis

The DNA of *E. coli* BL21(DE3) pLysS containing pRSET C-LipL32 was extracted using the QIAamp DNA Mini Kit (Qiagen GmbH, Hilden, Germany) with some modifications. Briefly, 500 μL of culture fluid was centrifuged at 16,000× *g* for 15 min. Subsequently, 200 μL of phosphate-buffered saline (PBS) was added to the supernatant and the samples were heated at 96°C for 10 min and processed according to the QIAamp DNA Mini Kit (Qiagen) procedure. The PCR analysis of LipL32 gen was performed using 5’-TTA CCG CTC GAG GTG CTT TCG GTG GTC TGC-3’ forward primer [24] and 5’-TGT TAA GAA TTC TTA CTT AGT CGC GTC AGA-3’ reverse primer [29]. PCR was performed using the KAPA HiFi HotStart ReadyMix PCR Kit (Roche, Indiana, USA). Thermocycler conditions were as follows: Initial denaturation at 95°C for 3 min, followed by 35 cycles of denaturation circuit at 98°C for 20 s, primary annealing at 65°C for 15 s, extension at 72°C for 24 s, and final extension at 72°C for 48 s. Electrophoresis was used to analyze the PCR results.

### Analysis of *E. coli* BL21(DE3) pLysS cultures containing pRSET C-LipL32 with sequencing

The PCR product from *E. coli* BL21(DE3) pLysS containing pRSET C-LipL32 was also sent to the first BASE DNA sequencing for analysis. The resulting sequence data were then analyzed using BioEdit software (https://bioedit.software.informer.com/7.2/) and contig sequence results in Blast to determine similarity with the *Leptospira*/OMP *Leptospira* (LipL32) bacterial sequences in GenBank.

### Expression and purification of the rLipL32 protein

The rLipL32 protein from *L. interrogans* serovar hardjo was produced and purified as described in the previous studies [30] with several modifications. Briefly, 500 mL of LB medium supplemented with 2% glucose was inoculated with 25 mL of *E. coli* BL21(DE3) pLysS culture containing pRSET C-LipL32. Cultures were grown at 37°C with shaking until OD_600_ = 0.4–0.6. The culture was then incubated with 1 mM isopropyl-beta-D-thiogalactopyranoside (IPTG) for 4 h at 37°C. We harvested the cells by centrifugation at 11,000× *g* for 15 min at 4°C. Purification of the rLipL32 protein under native conditions using a Ni-NTA matrix according to the QIAexpressionist procedure (Qiagen) was performed. We dialyzed and concentrated the eluted rLipL32 protein using Centriprep-30 (Millipore-Amicon, Beverly, MA). Protein purity of rLipL32 was analyzed by SDS-PAGE, visualized by Coomassie Brilliant Blue staining, and protein concentration was measured using a nanospectrophotometer.

### Production of anti-rLipL32 serum

Anti-rLipL32 sera were prepared from leptospirosis-free New Zealand rabbits weighing 2.5–3 kg and aged 10–16 weeks. Rabbits were divided into two treatment groups of three rabbits each. Before treatment, rabbits were bled to obtain serum and tested using MAT to confirm that the serum was negative for leptospirosis. In the first group, rabbits were injected subcutaneously with rLipL32 protein (150 μg/mL) mixed with Freund’s complete adjuvant (Sigma-Aldrich, USA) in a 1:1 ratio. The second and third immunizations were performed 4 and 8 weeks after the first immunization by injecting rLipL32 protein (150 μg/mL) mixed with Freund’s incomplete adjuvant (Sigma-Aldrich) 24]. The second group of rabbits was subcutaneously immunized with 150 μg/mL rLipL32 protein mixed with Montanide ISA 70 MVG (Seppic, Paris, France). Two weeks after the first immunization, the rabbits were again immunized with the same rLipL32 protein and adjuvant as before. Anti-rLipL32 rabbit serum samples were collected every 2 weeks for 3 months after the last immunization in each treatment group. Serum was analyzed using ELISA.

### ELISA for analysis of anti-rLipL32 serum

ELISA optimization was used to determine the concentration of rLipL32 protein, serum dilution, and conjugate to be used in the test. The rLipL32 protein concentrations for titration were 1, 2, 4, and 6 μg/mL, serum dilution was 1/200 and 1/400, and conjugate dilution was 1/10,000 and 1/20,000. The rLipL32 protein (2 μg/mL) was dissolved in coating buffer and incubated overnight at 4°C. Plates were blocked with 0.5% casein in PBS at room temperature (25^o^C) for 2 h. Plates were then washed 3 times with PBS containing 0.05% Tween 20. Anti-rLipL32 sera, positive, and negative control sera (diluted 1/400 in PBS containing 0.05% Tween 20 [PBST]) (100 μL) were added to the wells, and the plates were incubated at 25^o^C for 1 h. The plates were washed thrice with PBST and incubated with goat anti-rabbit IgG-HRP conjugate (dilution 1/20,000 in PBST) at 25^o^C for 1 h. Plates were washed again and 100 μL of TMB (Millipore) solution was added, and the plates were incubated for 5 min. Subsequently, 100 μL of 5% sulfuric acid was added to stop the reaction, and the absorbance of the reaction product was measured at 450 nm.

### Purification of IgG anti-rLipL32 antibody

Rabbit serum containing IgG was purified usingPureProteome™ Protein A/G Mix Magnetic Beads (Merck, Darmstadt, Germany) according to the kit procedure to obtain IgG anti-rLipL32 antibody. Immunoglobulin G was desalted and centrifuged at 4500× *g* for 30 min using Amicon Ultra 15 (Merck). The solution mentioned above was accommodated and was IgG.

### Analysis of protein and IgG anti-rLipL32 antibody using SDS-PAGE and Western blotting

Protein purification and IgG anti-rLipL32 antibody results were analyzed by SDS-PAGE using the TGX Stain-Free™ FastCast™ Acrylamide Kit, 12% (Bio-Rad, California, USA). Samples were prepared in a suitable volume with 2 × SDS loading buffer and heated at 100°C for 3 min. Samples (15 μL) were placed into the wells and run at 90 V for 1 h and 45 min. The gel was then stained with Coomassie Brilliant Blue. IgG anti-rLipL32 antibody was also analyzed using western blotting. Bacterial culture suspensions of *Leptospira* serovars icterohaemorrhagiae, bataviae, celledoni, javanica, canicola, rachmati, ballum, australis, pyrogenes, grippotyphosa, cynopteri, pomona, hardjo, and tarassovi, *E. coli*, and *S. aureus* (1.5 × 10^8^ cells/mL) were loaded into SDS-PAGE gel. Separate proteins were electro transferred to a nitrocellulose membrane. Membranes were washed 3 times with tris-buffered saline (TBS) buffer and incubated in blocking buffer (1% casein in TBS) for 1 h at 25^o^C. Membranes were washed 3 times with TBS-Tween buffer and incubated with IgG anti-rLipL32 antibody (5 μg/mL diluted in blocking buffer) for 1 h at 25^o^C. Membranes were washed 3 times with TBS-Tween buffer and incubated for 1 h at 25^o^C with goat anti-rabbit IgG-HRP conjugate (dilution 1/3000 in blocking buffer). The membranes were washed 3 times with TBS-Tween buffer and stained with 3,3′-diaminobenzidine substrate (0.007 g DAB, 7 μL H_2_O_2_, dissolved in 10 mL 60 mM Tris buffer, pH = 7.6).

## Results

### Bacterial culture of E. coli BL21 (DE3) pLysS containing pRSET C-LipL32 and analysis by PCR and sequencing

**Figure-1:**
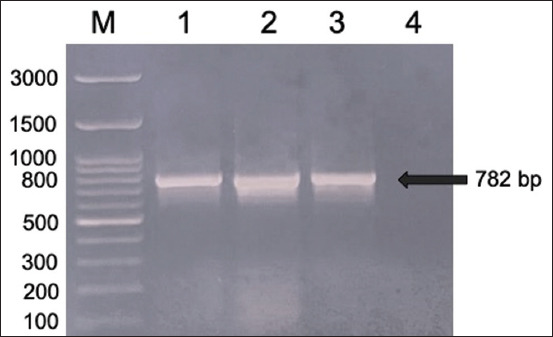
Electrogram of LipL32 gene amplification from *Escherichia coli* rLipL32 colony (M = marker; 1–2 = LipL32 gene from *E. coli* rLipL32 colony; 3 = LipL32 gene from *Leptospira interrogans* serovar Hardjo; 4 = Negative).

*E. coli* BL21 (DE3) pLysS containing pRSET C-LipL32 were cultured on LB medium, and the growing colonies were analyzed by PCR to confirm the presence of the LipL32 gene insert using specific LipL32 primers. The PCR product was analyzed by electrophoresis and the results are shown in Figure-1.

**Figure-2:**
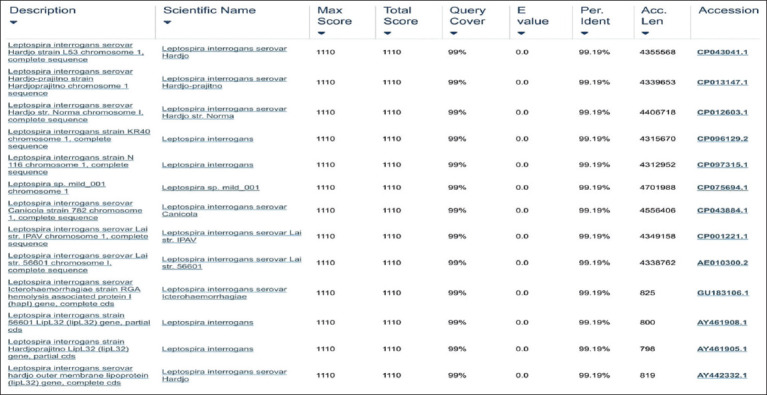
Sequencing analysis of polymerase chain reaction products from *Escherichia coli* rLipL32 colonies blasted with GenBank data available.

**Figure-3:**
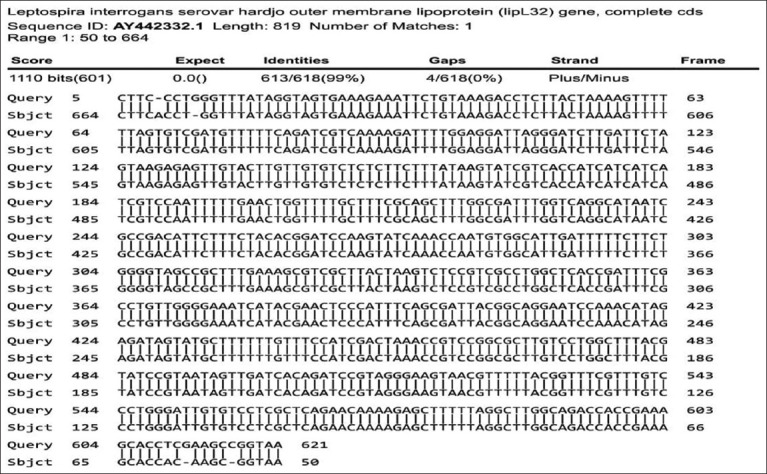
Sequencing of *Escherichia coli* rLipL32 colony polymerase chain reaction products compared to the outer membrane lipoprotein (LipL32) gene of *Leptospira interrogans* serovar hardjo.

Figure-1 shows that the PCR results can amplify the LipL32 gene from the *E. coli* BL21 (DE3) pLysS colony containing pRSET C-LipL32 and from the *L. interrogans* serovar hardjo as a positive control with an amplicon size of 782 bp compared to the marker used. Sequencing analysis of the LipL32 gene from *E. coli* BL21 (DE3) pLysS containing pRSET C-LipL32 was also performed, and the results are presented in Figures-2 and 3.

Figures-2 and 3 show sequence analysis of the PCR product of the *E. coli* BL21 (DE3) pLysS colony containing pRSET C-LipL32, which has 99.19% identity with the *L. interrogans* serovar hardjo, canicola, lai, and icterohaemorrhagiae chromosome 1 sequence, as well as with the outer membrane lipoprotein (LipL32) of *L. interrogans* serovar hardjo compared with data in GenBank.

### Expression and purification of rLipL32 protein and analysis with SDS-PAGE

**Figure-4:**
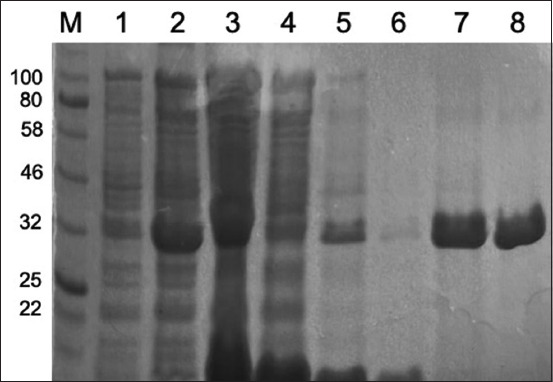
Sodium dodecyl sulfate-polyacrylamide gel electrophoresis results of the rLipL32 protein purification stage (M = Marker; 1 = Uninduced cell; 2 = Induced cell; 3 = Supernatant of lysed cell; 4 = Flow-through fraction; 5 = Wash 1; 6 = Wash 2; 7 = LipL32 elution fraction; 8 = Concentrated LipL32 protein).

Colonies of *E. coli* BL21 (DE3) pLysS containing pRSET C-LipL32 that were confirmed to contain the LipL32 gene were used to express and purify rLipL32 protein at a later stage. Bacteria were cultured in liquid LB medium, and after reaching an OD600 of 0.545, cells were induced with 1 mM IPTG for 4 h, and His-tagged protein was purified under native conditions using the QIAexpress® Ni-NTA Fast Start Kit (Qiagen). The protein purification stages were characterized using SDS-PAGE and are presented in Figure-4.

The analysis results of the protein purification process using SDS-PAGE (Figure-4) indicated that the elution fraction contained recombinant LipL32 protein, which was characterized by a band measuring 32-kDa (columns 7 and 8) compared to the marker used. These results indicate that *E. coli* BL21 (DE3) pLysS containing pRSET C-LipL32 expresses the LipL32 fusion protein. The concentration of purified protein in the eluted fraction was measured using a nanospectrophotometer, and the result was 1.27 mg/mL.

### Production and analysis of anti-rLipL32 sera using ELISA

**Figure-5:**
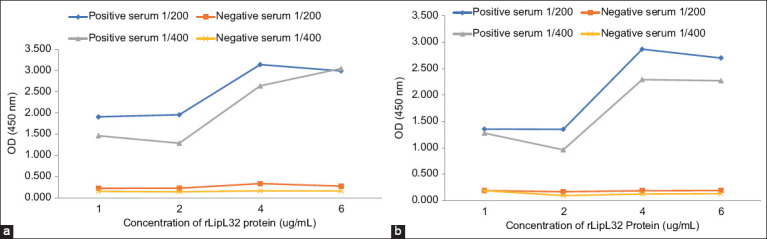
Enzyme-linked immunosorbent assay optimization results with checkerboard titration for rLipL32 protein concentrations of 1, 2, 4, and 6 μg/mL; dilution of serum 1/200, and 1/400; (a) conjugate 1/10,000 and (b) conjugate 1/20,000.

The purified rLipL32 protein was then used to produce anti-rLipL32 serum in rabbits using Freund’s adjuvant and Montanide ISA 70 MVG. The collected anti-rLipL32 serum was then analyzed using ELISA. Before the analysis of anti-rLipL32 serum, ELISA optimization was performed with checkerboard titration to determine optimal conditions of rLipL32 protein concentration for coating, serum dilution, and conjugate used in the test. ELISA optimization results are shown in Figure-5.

**Figure-6:**
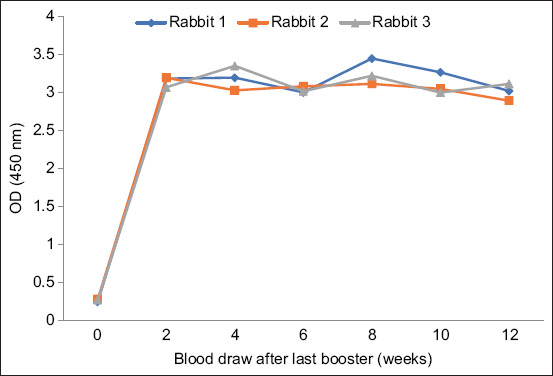
Anti-rLipL32 serum analysis of rabbits immunized with rLipL32 protein and Freund’s adjuvant.

**Figure-7:**
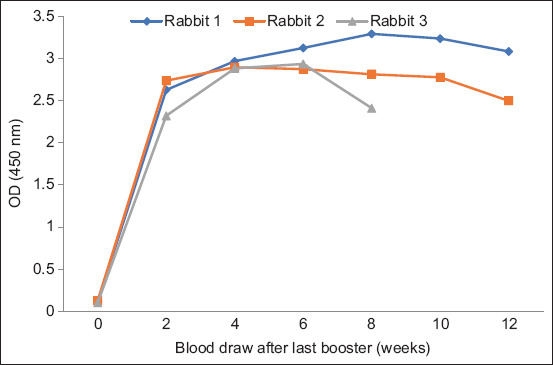
Analysis of anti-rLipL32 serum from rabbits immunized with the rLipL32 protein and Montanide ISA 70 MVG adjuvant.

Based on Figure-5, the ELISA optimization results used for anti-rLipL32 serum analysis showed a P/N ratio of 10.223 at rLipL32 protein concentration of 2 μg/mL, a serum dilution of 1/400, and a conjugate dilution of 1/20,000. These optimal conditions were then used for analysis of serum anti-rLipL32. Figures-6 and 7 show the results of the ELISA analysis of anti-rLipL32 serum produced using Freund’s adjuvant and Montanide ISA 70 MVG.

As shown in Figures-6 and 7, the ELISA results showed a high increase in the OD values in anti-rLipL32 serum produced using Freund’s adjuvant and Montanide ISA 70 MVG at 2–12 weeks after the last booster compared with preimmune serum. The production of anti-rLipL32 serum using ISA 70 MVG montanide adjuvant was shorter than that using Freund’s adjuvant. Rabbit 3 on anti-rLipL32 serum, which was immunized with rLipL32 protein and Montanide is a 70 MVG adjuvant, died at 9 weeks after the last booster; therefore, blood sampling can only be performed up to 8 weeks after the last booster.

### Purification of IgG anti-rLipL32 antibody

**Figure-8:**
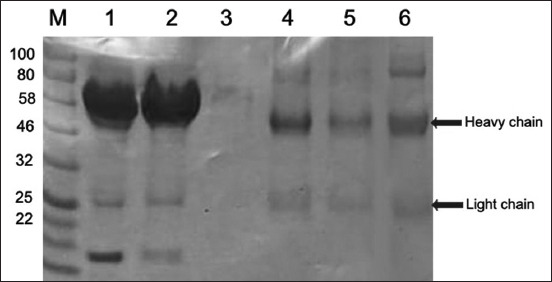
Sodium dodecyl sulfate-polyacrylamide gel electrophoresis results of immunoglobulin G (IgG) anti-rLipL32 antibody purification steps (M = Marker; 1 = Rabbit serum; 2 = Flow-through; 3 = wash; 4 = Elution 1 (IgG); 5 = Elution 2 (IgG); 6 = IgG concentration).

The collected rabbit anti-rLipL32 serum was purified to produce anti-rLipL32 IgG and analyzed using SDS-PAGE. Figure-8 shows the IgG anti-rLipL32 purification stage analysis results.

Analysis of the purification stages of the IgG anti-rLipL32 antibody using SDS-PAGE (Figure-8) revealed two bands showing a heavy chain with a molecular weight in the range of 50 kDa and a light chain in the range of 25 kDa compared to the markers used.

### Analysis of IgG anti-rLipL32 antibody by western blotting

**Figure-9:**
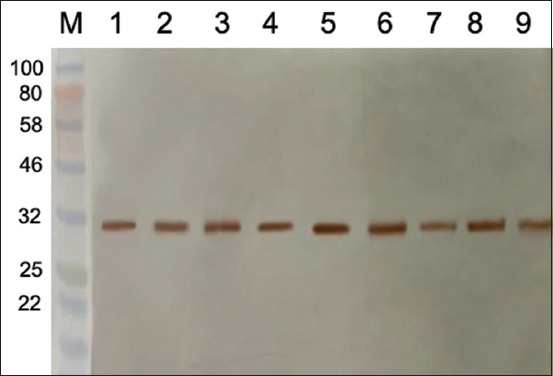
Results of western blotting analysis of immunoglobulin G anti-rLipL32 with *Leptospira interrogans* serovar protein: 1 = Icterohaemorrhagiae; 2 = Javanica; 3 = Celedoni; 4 = Canicola; 5 = ballum; 6 = Pyrogenes; 7 = Cynopteri; 8 = Rachmati; 9 = Australia; M = markers.

**Figure-10:**
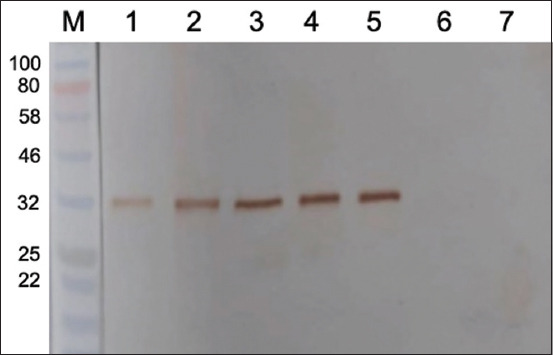
Results of western blotting analysis of immunoglobulin G anti-rLipL32 with *Leptospira interrogans* serovar protein: 1 = Pomona; 2 = Grippothyphosa; 3 = Hardjo; 4 = Batavia; 5 = Tarrasovi; 6 = *Escherichia coli*; 7 = *Staphylococcus aureus*; M = Markers.

Western blotting was performed to evaluate the binding ability of IgG anti-rLipL32 antibodies to target proteins in pathogenic *Leptospira*. Bacterial cultures of *Leptospira* serovars icterohaemorrhagiae, bataviae, javanica, ballum, celledoni, pyrogenes, canicola, cynopteri, australis, rachmati, pomona, tarassovi, hardjo, grippotyphosa, *E. coli*, and *S. aureus* were used in western blotting analysis. Western blot analysis results are shown in Figures-9 and 10.

Western blotting analysis (Figures-9 and 10) showed that the IgG anti-rLipL32 antibody binds specifically to the *Leptospira* serovar used (at 32 KDa) but does not bind to *E. coli* and *S. aureus*.

## Discussion

Leptospirosis continues to jeopardize the health of humans, livestock, and wild animals, causing economic losses and death. Diagnosis of the disease is usually based on serological testing with MAT; however, this test uses live cultures of various *Leptospira* serovars and specialized personnel, posing a challenge to field application [31]. Serological tests using recombinant antigens have higher sensitivity and specificity compared to whole bacterial cells because of the purity of the antigen and the reduction of non-specific parts in whole bacterial cells [32]. Purified antigen is considered to be a key factor in the diagnosis of leptospirosis. A good antigen should be expressed only on pathogenic *Leptospira* *spp*., have a basic target on the immune response, be able to distinguish leptospirosis from other diseases, and be conserved among over 200 *Leptospira* serovars [29].

The leptospiral OMP component is an essential approach for the development of alternative leptospirosis diagnostic techniques. Several *Leptospira* proteins, such as LipL32, LigA, OmpL1, and LipL21, have been used as antigens for antibody detection [10]. Among the OMPs, LipL32 is widely used as a biomarker for antigen detection and is a potential candidate for vaccine and therapy production [27]. Therefore, this protein can be used in the development of promising diagnostic tools [33]. The LipL32 protein is a highly conserved *Leptospira*-specific antigen [34] and has great diagnostic potential for the detection of leptospirosis by ELISA [35]. In this study, rLipL32 protein production was performed to produce and characterize IgG anti-rLipL32 as an antigen source for developing leptospirosis diagnostic techniques.

In the analysis of *E. coli* rLipL32 colonies by PCR (Figure-1), the forward primer was attached to the 50^th^ base sequence of the start codon, whereas the reverse primer was attached to the 831^st^ base (12 bases after the stop codon) of the LipL32 gene nucleotide sequence, *L. interrogans* serovar hardjo, obtained from GenBank with accession number AY442332.1, so the size of the DNA obtained was 782 bp. In a previous study, PCR analysis of the LipL32 gene, which was cloned into the pRSET B vector and transformed into *E. coli* BL21 (DE3), produced an amplicon size of 720 bp [33]. The LipL32 protein is highly conserved among pathogenic *Leptospira* species and is expressed during infection [33]. Sequencing analysis of *E. coli* rLipL32 colony PCR products (Figures-2 and 3) showed 99.19% identity with several pathogenic *Leptospira* serovars and *Leptospira* OMP (LipL32). In a previous study, sequence analysis of the LipL32 gene produced from *L. interrogans* serovar pomona aligned to the P1 and P2 sequences and showed 100% homology to both peptides [30].

*E. coli* rLipL32 colonies were used to produce and purify the rLipL32 protein, and the protein purification steps were analyzed using 12% SDS-PAGE (Figure-4). SDS-PAGE analysis indicated that the rLipL32 protein was expressed in a 32-kDa band. The findings of this research support previous studies showing that the rLipL32 protein appears as the main band on SDS-PAGE after purification with the Ni-NTA resin purification system [33]. A high-intensity band at 32-kDa was observed when the purification fraction was analyzed by SDS-PAGE [36]. Using SDS-PAGE, a protein with a molecular weight of approximately 32-kDa was found to be the most dominant band in the total protein profile of *Leptospira* [37]. LipL32, the main OMP of *Leptospira*, is expressed only in pathogenic *Leptospira* species [24]. Protein concentration was measured using a NanoSpectrophotometer and the result was 1.27 mg/mL. Previous studies have shown that the purification of rLipL32 protein by affinity chromatography was quite efficient and yielded protein concentrations of 1 mg/mL [36], 0.518 mg/mL [29], and 3.1 mg/mL [32].

Purified rLipL32 protein produced anti-rLipL32 serum in rabbits using Freund’s adjuvant and Montanide ISA 70 M VG. The produced anti-rLipL32 serum was then analyzed using ELISA. The optimum concentration of rLipL32 protein, serum dilution, and conjugate dilution were determined by checkerboard titration. The resulting P/N ratio (positive serum OD/serum negative OD) used in the test was 10.223 at rLipL32 protein concentration of 2 μg/mL, serum dilution of 1/400, and conjugate dilution of 1/20,000 (Figure-5). This P/N value is determined on the basis of the highest value resulting from the P/N ratio of the lowest concentration of rLipL32 protein, the highest dilution of serum, and conjugate for the minimum use of antigen, serum, and conjugate. The higher the P/N ratio, the easier, and clearer it is to differentiate and determine positive and negative sera [38].

The results of ELISA analysis of anti-rLipL32 serum immunized with Freund’s adjuvant and Montanide ISA 70 M VG showed an increase in the OD value compared with pre-immunize serum (Figures-6 and 7). The use of an adjuvant Montanide ISA 70 M VG tends to show stable OD values at different collection times, and the time needed to produce anti-rLipL32 serum is relatively shorter compared to the Freund adjuvant. The previous studies have also shown that the use of Montanide adjuvants is easier, reduces animal inflammation and discomfort, and has fewer side effects. Freund’s adjuvant is thicker than montanide, making injection more difficult [39, 40].

Anti-rLipL32 serum produced in rabbits was then purified to obtain IgG anti-rLipL32 antibodies, and IgG anti-rLipL32 purification stages were analyzed using 12% SDS-PAGE. SDS-PAGE analysis showed the presence of two bands, the heavy chain with a molecular weight in the range of 50 kDa and the light chain with a molecular weight in the range of 25 kDa (Figure-8). Polyclonal antibodies against LipL32 were produced using three peptide chains (EP3, EP4, and EP6), which were chemically synthesized and injected into male New Zealand rabbits, and serum was used for immunomagnetic separation test without purification [13].

The produced anti-rLipL32 IgG was characterized by western blotting using different pathogenic *Leptospira* serovars, *E. coli*, and *S. aureus* bacteria to identify whether they specifically reacted with the rLipL32 protein. From the western blotting results (Figures-9 and 10), a band at 32 KDa was observed when IgG anti-rLipL32 antibody was reacted with various pathogenic *Leptospira* serovars, whereas no band was observed when IgG anti-rLipL32 antibody was reacted with *E. coli* and *S. aureus* bacteria. The produced IgG anti-rLipL32 antibody showed good reactivity with various pathogenic *Leptospira* serovars tested, and no reaction was observed when IgG anti-rLipL32 was reacted with *E. coli* and *S. aureus*. These results are consistent with the previous studies of anti-rLipL32 antibodies produced using three peptide chains (EP3, EP4, and EP6), which showed specific immunoreactivity in the main band with a molecular size of 32-kDa in all *Leptospira* strains used [13]. Anti-rLipL32 antibody has also been used in a nanogold-based dot-blot immunoassay and proved to be a sensitive, rapid, and reliable method for early leptospirosis detection [41].

## Conclusion

The IgG anti-rLipL32 antibody binds and cross-reacts with pathogenic *Leptospira* bacteria in the same region of the rLipL32 molecule but does not bind to *E. coli* and *S. aureus* bacteria. IgG anti-rLipL32 antibodies are highly specific and sensitive against *Leptospira* pathogens. These results indicate that IgG anti-rLipL32 antibody can be used as a promising biomarker for diagnosing leptospirosis.

## Authors’ Contributions

SS: Conceptualized, designed the study, collected and analyzed data, and drafted the manuscript. PPS: Conceptualized, designed the study and drafted the manuscript. NLPID and PAY: Designed the study and analyzed data. All the authors have read, reviewed, revised, and approved the final manuscript.
